# Efficient delivery of small RNAs to podocytes in vitro by direct exosome transfection

**DOI:** 10.1186/s12951-025-03426-7

**Published:** 2025-05-23

**Authors:** Tim Lange, Luzia Maron, Claudia Weber, Doreen Biedenweg, Rabea Schlüter, Nicole Endlich

**Affiliations:** 1 Institute of Anatomy and Cell Biology, University Medicine Greifswald, Friedrich-Loeffler-Str. 23c, 17487 Greifswald, Germany; 2 Institute for Physics, University of Greifswald, Greifswald, Germany; 3 Imaging Center of the Department of Biology, Greifswald of University, Greifswald, Germany

**Keywords:** Podocyte, Exosomes, Transfection, Vesicles, CKD, Small RNA, MiRNA, SiRNA

## Abstract

**Background:**

Podocytes are a crucial component of the glomerular filtration barrier, and changes in their 3D structure contribute to over 80% of chronic kidney disease (CKD) cases. Exosomal small RNAs play a key role in cell–cell communication in CKD and may serve as nanocarriers for delivering small RNAs into podocytes. However, the uptake of exosomal cargo by podocytes remains poorly understood. This study explores the use of isolated exosomes, directly transfected with fluorescently-labeled small RNAs, for tracking and delivering small RNAs to cultured podocytes.

**Methods:**

Exosomes were isolated from immortalized murine podocytes and transfected with Cy3-labeled siRNA and miRNA controls using the ExoFect siRNA/miRNA Transfection Kit. We characterized the transfected exosomes via transmission electron microscopy (TEM) and Western blot for exosomal markers CD9 and TSG101. Subsequently, we co-cultured these exosomes with podocytes and used confocal laser-scanning microscopy (cLSM), and structured illumination microscopy (SIM) to visualize cargo uptake, confirmed through flow cytometry, imaging flow cytometry and immunofluorescence staining for Rab5, Rab7, and CD9. The isolated exosomes were also transfected with pre-miR-21 and filamin A (FlnA)-siRNAs before being co-cultured with podocytes. We confirmed the efficiency of transfection and knockdown using RT-qPCR, Western blotting, and immunofluorescence staining.

**Results:**

TEM revealed that the exosomes maintained a consistent shape and size of approximately 20 nm posttransfection and exhibited a stable expression of CD9 and TSG101. Flow cytometry and immunofluorescence imaging showed that podocytes take up Cy3-labeled exosomal miRNAs and siRNAs time-dependently, utilizing various mechanisms, including encapsulation within vesicular structures, endocytosis and free distribution within the cells. Transfection of exosomes with FlnA-siRNAs resulted in a significant 2.8-fold reduction of filamin A expression in co-cultured podocytes, while pre-miR-21-transfected exosomes led to a remarkable 338-fold increase in mature miR-21 levels.

**Conclusions:**

These findings demonstrate that direct exosome transfection with fluorescently-labeled small RNAs is an effective method for tracking exosomal cargo in podocytes. This study is the first to show that directly transfected exosomes can deliver small RNAs to podocytes in vitro, suggesting their potential as RNA carriers for therapeutic strategies in more complex settings.

**Graphical Abstract:**

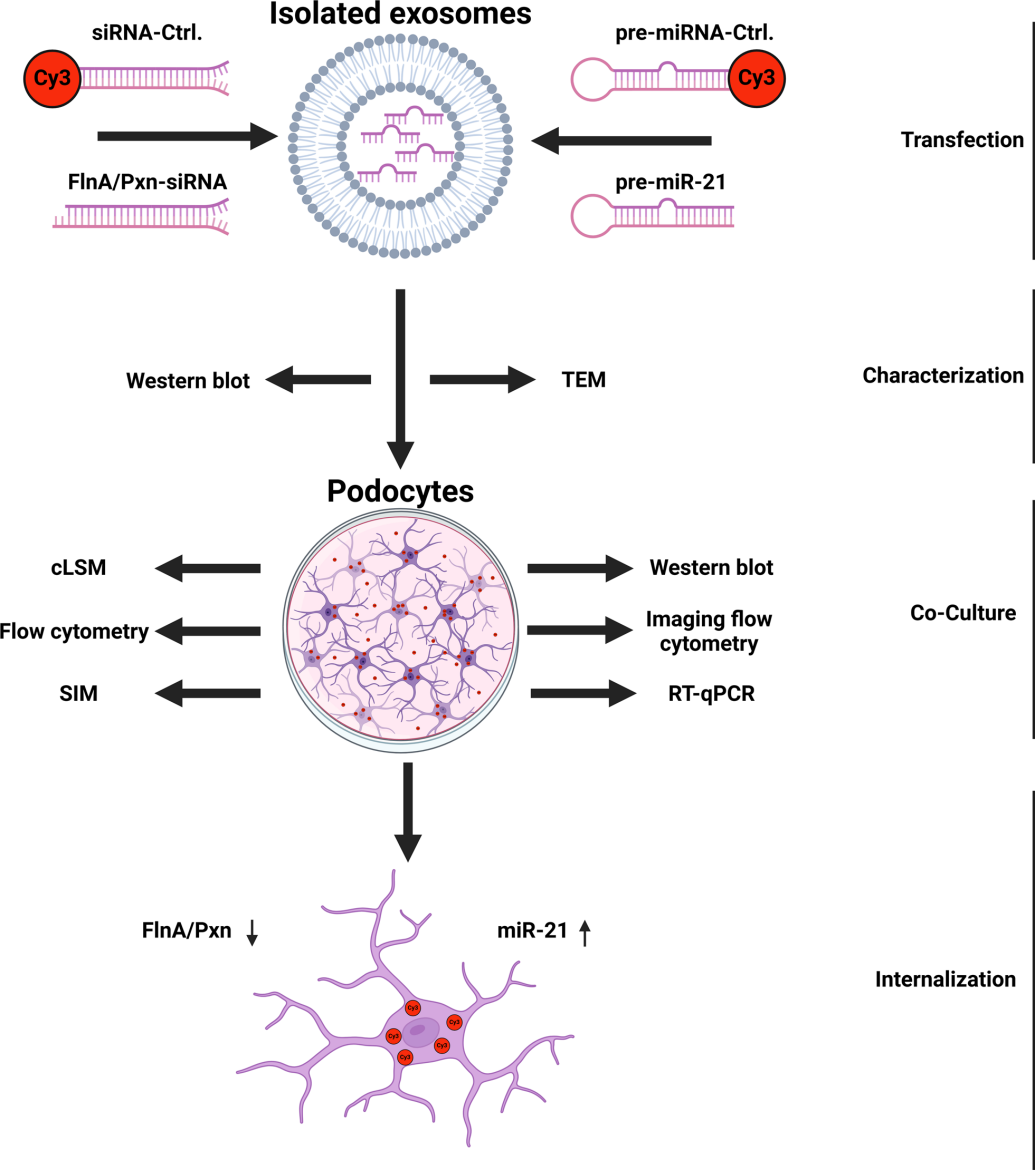

**Supplementary Information:**

The online version contains supplementary material available at 10.1186/s12951-025-03426-7.

## Introduction

Podocytes, a highly specialized renal epithelial cell type, cover the outer aspect of the glomerular capillaries and are crucial for the size-selectivity of the filtration barrier. Changes in the complex 3D morphology of their interdigitating foot processes or their loss are the leading causes of 80% of chronic kidney diseases (CKDs) [1].

CKD is a major public health burden affecting more than 10% of the world’s population [2]. Until now, there has been no causal therapy for the treatment of CKD patients [2]. If CKD progresses, renal replacement therapies such as dialysis and transplantation are necessary. However, they are often associated with significant limitations in quality of life and with increased mortality. Therefore, advanced strategies to generate new treatment options are urgently needed.

The study of extracellular vesicles, particularly exosomes, is an innovative and promising approach for the treatment of CKD. These vesicles play important roles in cell‒cell communication between podocytes and can also act as natural carriers for a variety of molecular cargos, such as proteins, metabolites, DNA, RNA and small RNAs [3]. Podocytes release increased amounts of exosomes containing disease-specific small RNA cargo during kidney injury [4], especially miRNAs, which have recently come into the focus of kidney research [5, 6]. Furthermore, increasing evidence suggests that exosomes could also function as delivery vehicles for small RNAs to podocytes. This would open up completely new avenues for therapeutic interventions [7].

Current studies primarily use indirect loading strategies, such as the use of exosomes isolated from naturally overexpressing cell types like stem cells, to treat podocyte-related diseases [8]. Another common approach involves the transfection of donor cells with specific small RNAs and the subsequent isolation of the enriched exosomes that they secrete [9]. Both methods have major disadvantages: they lack specificity due to the presence of other endogenous small RNAs, they often have insufficient intracellular packaging of the target small RNAs, they have reduced reproducibility, and the transfection reagents used are difficult to remove and often toxic to recipient cells [10].

A more promising approach than the currently preferred indirect methods could be the direct transfection of exosomes with small RNAs. Direct transfection of exosomes enables precise cargo loading with higher efficiency, consistency, and scalability, bypassing donor cell dependence and allowing incorporation of non-endogenous materials for therapeutic applications. However, researchers have not yet determined whether cultured podocytes can effectively internalize these cargo-loaded exosomes and whether the small RNAs contained in these exosomes remain functional. To address these questions, we directly transfected exosomes with fluorescentlylabeled miRNAs and siRNAs, demonstrating their potential as a viable tool for delivering small RNAs into podocytes.

## Materials and methods

### Cell culture

Conditionally immortalized podocytes [11] (SVI; NIPOKA GmbH, Greifswald, Germany) were used for all the cell culture experiments and were handled as previously described [12]. Podocytes were maintained in RPMI 1640 medium (Sigma‒Aldrich, St. Louis, MO, USA) supplemented with 10% fetal bovine serum (FBS; Boehringer Mannheim, Mannheim, Germany), 100 U/mL penicillin, and 0.1 mg/mL streptomycin (Thermo Fisher Scientific, Waltham, MA, USA). For expansion, podocytes were cultured at 33°C and 5% CO_2_. To induce podocyte differentiation, podocytes were cultured at 38°C and 5% CO_2_ for at least two weeks before the experiments. Prior to exosome isolation, the cells were washed three times with Dulbecco’s PBS (PBS, Sigma‒Aldrich), and the medium was replaced with RPMI 1640 supplemented with 10% exosome-depleted FBS (System Biosciences, Palo Alto, CA, USA), 100 U/mL penicillin and 0.1 mg/mL streptomycin.

### Exosome isolation

The cells were cultured in exosome-depleted media for 3 days. The conditioned medium was transferred to a 15 mL conical tube and centrifuged for 15 min at 3000×*g* at room temperature (RT) to remove cells and cell debris. The supernatant was transferred to a fresh 15 mL conical tube and centrifuged again for 15 min at 3000×*g* at RT to remove residual cell debris. Afterwards, 2 mL of Exoquick TC (System Biosciences) was added to the supernatant. After inverting the tube several times, it was incubated at 4°C overnight. Afterwards, the supernatant was centrifuged for 1 h at 10,000×*g* at 4°C. The exosome pellet was eluted in RPMI 1640 medium without supplements.

### Exosome transfection

Exosome transfection was performed with the Exo-Fect™ siRNA/miRNA Transfection Kit (System Biosciences) following the manufacturer’s instructions with some modifications. We used the Silencer Select Flna siRNA (#s101260, Ambion, Thermo Fisher Scientific), Silencer Select Pxn siRNA (#s72562, Ambion, Thermo Fisher Scientific), pre-miR™ miRNA Precursor miR-21 mimics (#AM17100, Ambion, Thermo Fisher Scientific) and the corresponding negative controls (Silencer™ Cy3™-labeled Negative Control No. 1 siRNA, Silencer™ Negative Control No. 1 siRNA and Cy3™-labeled pre-miR Negative Control #1, Ambion, Thermo Fisher Scientific). The volume of the transfection reaction was 110 µL and included 545 nM of each nucleic acid. After incubation for 15 min at RT, the transfection reaction mixture was applied to 100 µL of the eluted exosomes or PBS as a negative control, respectively. This mixture was incubated for 1 h at 37°C in the dark. Exosome cleanup was performed according to the manufacturer’s instructions.

### Exosome treatment

Prior to exosome treatment, differentiated podocytes were washed twice with 10 mL of PBS, and the cells were dissociated with 3 mL of trypsin–EDTA (0.05%, 0.02%, Thermo Fisher Scientific). The trypsin–EDTA reaction was blocked by the addition of 9 mL of RPMI 1640 supplemented with exosome-depleted FBS, 100 U/mL penicillin and 0.1 mg/mL streptomycin. The cells were subsequently pelleted via centrifugation at RT and 750×*g* for 5 min. The pellet was subsequently eluted in 1 mL of RPMI 1640 supplemented with exosome-depleted FBS, 100 U/mL penicillin and 0.1 mg/mL streptomycin. After cell counting, 2.5 × 10^4^ cells per well were seeded in each well of a collagen 4-coated (Thermo Fisher Scientific) 6-well plate in 2 mL of RPMI 1640 supplemented with exosome-depleted FBS, 100 U/mL penicillin and 0.1 mg/mL streptomycin. The cells were subjected to exosome treatments after 3 days. The cleaned-up exosome elutions from the transfection experiments were applied to the corresponding wells. Cells were treated for 48 h for uptake- and knock down experiments and for different durations (4 h to 1 week) and concentrations (18.75–150 µg) to characterize uptake dynamics.

### Protein isolation and Western blot

Protein isolation from cells was performed as previously described [13, 14]. Prior to protein isolation, the cells were washed 3 times with 2 mL of PBS.

For protein isolation from the transfected and untransfected exosomes, 100 µL of exosome mixture was supplemented with 20 µL of Exoquick TC, inverted and incubated on ice for 1 h. Afterwards, the samples were centrifuged for 1 h at 14,000×*g* and 4 °C. The pellets were eluted in Pierce IP Lysis Buffer (500 mM, Thermo Fisher Scientific). Further protein isolation and concentration determination via the Bradford assay were performed as previously described [13, 14].

The samples were subsequently adjusted to 20 μg/lane (TSG101) and 10 μg/lane (CD9) for qualitative analysis. For quantitative analysis, we used 20 µL of each exosome sample per lane. For the analysis of cultured podocytes after treatment with FlnA-siRNA exosomes, we adjusted all the samples to 2 µg per lane. For the comparison of cultured podocytes and isolated exosomes we used 30 µg per lane. All samples were mixed with 6× sample buffer (0.35 M Tris [pH 6.8], 0.35 M SDS, 30% v/v glycerol, 0.175 mM bromophenol blue) and boiled at 95°C for 5 min. The protein samples were separated on a 4–20% gradient Mini-Protean TGX gel stain-free (Bio-Rad, Hercules, CA, USA). The separated proteins were blotted on nitrocellulose membranes via the Trans-Blot Turbo RTA Transfer Kit (Bio-Rad) and the Trans-Blot Turbo Transfer System (Bio-Rad) at 2.5 A/25 V for 5 min. Membranes were washed in 1× TBS + T wash buffer (50 mM Tris, 150 mM NaCl, 10 mM CaCl_2_, and 1 mM MgCl_2_ supplemented with 0.1% Tween-20; AppliChem, Darmstadt, Germany) and blocked in wash buffer supplemented with 5% milk powder (blocking solution) for 1 h at room temperature. The primary antibodies were diluted in blocking solution and incubated with the membranes overnight. After being washed 3 × 5 min with wash buffer, the membranes were incubated with secondary antibodies for 45 min, washed again for 4 × 5 min with wash buffer, developed with the ECL Prime Western Blotting Detection Reagent (Cytiva Europe GmbH, Freiburg, Germany) and visualized on X-ray films (Cytiva Europe GmbH) by using Carestream Kodak autoradiography GBX developer/fixer solutions. For normalization and usage of alternative antibodies on the same blot, the blots were stripped. The following antibodies were used at the final concentrations: anti-TSG101 (Sigma‒Aldrich; 1:1000), anti-CD9 (Thermo Fisher Scientific, 1:2000), anti-FlnA (Sigma‒Aldrich; 1:4000), anti-Gapdh (Santa Cruz Biotechnology, Santa Cruz, USA; 1:2000), and secondary anti-rabbit HRP (Santa Cruz Biotechnology; 1:6000 for TSG101; 1:5000 for CD9; 1:15,000 for FlnA and Gapdh).

### Transmission electron microscopy

For transmission electron microscopy (TEM), 10 mL of cell- and debris-free cell culture media were treated with ExoQuick-TC (System Biosciences) for exosome isolation according to the manufacturer’s instructions with minor modifications. To 10 mL of cell culture media, 3.3 mL of ExoQuick-TC was added, and the mixture was stored overnight at 4°C. Then, the samples were centrifuged at 10,000×*g* for 60 min, and the supernatant was discarded. After treatment the pellets were prepared for TEM according to Asadi and coworkers [15] via the flotation method for the staining procedure. Briefly, isolated exosomes were fixed with 2% paraformaldehyde in 0.1 M sodium phosphate buffer (pH 7.5) and then allowed to adsorb onto a glow-discharged carbon-coated porous Pioloform film on a 400-mesh grid (Plano GmbH) for 20 min on ice. The grid was then transferred onto four droplets of deionized water on ice for 2 min each and finally onto a drop of staining mixture for 10 min on ice. To prepare the staining mixture, 100 µL of 3% aqueous uranyl acetate was added to 900 µL of 2% methylcellulose, followed by the addition of 75 µL of deionized water and 25 µL of 1% phosphotungstic acid (pH 7). After blotting with filter paper, the grids were air-dried. All the samples were examined with a LEO 906 transmission electron microscope (Carl Zeiss Microscopy Deutschland GmbH, Oberkochen, Germany) at an acceleration voltage of 80 kV. For acquisition of the images at 100,000× magnification, a wide-angle dualspeed CCD camera Sharpeye (Tröndle, Moorenweis, Germany) was used, operated by the ImageSP software. All micrographs were edited by using Adobe Photoshop CS6.

### RNA isolation

For the miR-loading experiments, the cells were cultured for 24 h in the presence of transfected or untransfected exosomes or control exosomes as indicated. For the siRNA-loading experiments, the cells were treated for 48 h with the transfected exosomes or control exosomes as indicated. RNA isolation was performed as previously described [13]. Prior to the administration of Tri-reagent, the cell layer was washed twice with 2 mL of PBS.

### Taqman reverse transcription

cDNA synthesis was performed starting with 10 ng of total RNA via Taqman™ miRNA Assays and the Taqman™ miRNA Reverse Transcription Kit (Thermo Fisher Scientific). The following Taqman™ miRNA assays were used: Hsa-miR-21-5p: ID #000397 and U6 snRNA: ID #715680. The RT-reactions were performed according to the manufacturer’s instructions. The negative controls included no template- and no reverse transcriptase controls.

### Taqman qPCR

qPCR was performed with the Taqman™ miRNA Assays mentioned above and the Taqman™ Universal Master Mix II without UNG (Thermo Fisher Scientific) following the manufacturer’s instructions. The reaction mixtures contained 1.33 μL of undiluted cDNA solution and 18.67 μL of Master Mix. qPCR was performed on a Bio-Rad iQ5 thermal cycler (Bio-Rad) with the following cycling scheme: 10 min at 95°C followed by 45 cycles of 15 s at 95°C and 60 s at 60°C. All samples were run in triplicate. The negative controls included the no template controls from cDNA synthesis and an extra no template control for the qPCR. Ct values were calculated with automatically set thresholds and baselines via cycler software (Bio-Rad). Raw Ct values ≥ 38 were excluded from the analysis. All Ct values were normalized against U6 and the Cy3 w/o exosome Ctrl.

### Immunofluorescence staining

Prior to immunofluorescence staining, the cells were fixed with 2% paraformaldehyde (PFA) for 10 min and permeabilized with 0.3% Triton-X (Sigma‒Aldrich) for 3 min. Then, the cells were blocked for 1 h with blocking solution (2% FBS, 2% BSA and 0.2% fish gelatin in PBS). The primary antibodies were diluted 1:100 in blocking solution and incubated for 1 h at RT. We used the following primary antibodies: anti-FlnA (Sigma‒Aldrich), anti-CD9 (BD Biosciences, San Jose, CA), anti-Rab5 (Cell Signaling Technology, Leiden, Netherlands) and anti-Rab7 (Cell Signaling Technology). The following secondary antibodies were diluted 1:300 in blocking solution and incubated with the cells for 1 h: anti-mouse-Cy2 (Jackson ImmunoResearch Laboratories, Ely, UK) and anti-rabbit-Cy2 (Jackson ImmunoResearch Laboratories). The actin cytoskeleton was visualized by staining with Alexa Fluor 647 phalloidin (1:100; Thermo Fisher Scientific) for 1 h, which was subsequently added to the secondary antibody solution. For nuclear staining, DAPI diluted 1:100 in PBS (Sigma‒Aldrich) was used for 2 min. All the samples were mounted in Mowiol (Carl Roth, Karlsruhe, Germany).

### Confocal laser scanning microscopy (cLSM)

Confocal laser scanning microscopy (cLSM) was performed on a Leica TCS SP5 confocal microscope (Leica Microsystems, Wetzlar, Germany) with 20×, 40× and 63× oil immersion objectives. For image acquisition, Leica Application Suite software (Leica Microsystems, Version 2.6.0) was used. All z-stacks were acquired throughout the whole z-plane (20 µm) in 0.5 µm steps. Pre-miR-21 experiments were performed in 20 × 1 µm steps. The xzy-projection was acquired in 20 × 1.73 µm steps. For 3D-SIM, a Zeiss Elyra PS.1 A system (Zeiss Microscopy, Oberkochen, Germany) equipped with a 63 × oil immersion objective was used. Z-Stacks with a size of 1280 µm × 1280 µm and a slice-to-slice distance of 0.43 µm were acquired over approximately 2 µm using a 561 nm laser (3.5% laser power, exposure time: 150 ms), a 488 nm laser (3.5% laser power, exposure time: 150 ms) and a 405 nm laser (7% laser power, exposure time: 100 ms). The grating was shifted and rotated five times on every frame while widefield images were acquired. Z-stacks were acquired in 4 planes of 0.43 µm. 3D-SIM reconstruction was performed with Zeiss ZEN Black. ZEN blue software was used for maximum intensity projections.

### Flow cytometry and imaging flow cytometry

For flow cytometry, three independent replicates were processed including untreated podocytes, podocytes treated with exosomes without Cy3-miRCtrl that went through the transfection process as controls and Cy3-miRCtrlExos. Untreated podocytes were used as reference. Prior to flow cytometry, 300,000 cells of each treatment group were treated as described above for 48 h. Afterwards cells were washed twice with PBS and detached with Trypsin–EDTA for 5 min. The detached cells were diluted in RPMI 1640 supplemented with exosome-depleted FBS, 100 U/mL penicillin and 0.1 mg/mL streptomycin. Subsequently, the cells were centrifuged for 3 min at 750×*g* at RT. The pellet was washed with PBS and centrifuged again at the same conditions. After discarding the supernatant, the cells were fixed by the addition of PFA (2%) for 15 min. Finally, the cells were washed twice with PBS and resuspended in 500 µL PBS, stored on ice and transferred to flow cytometry. Flow cytometry was performed as previously described on an Aria III, Canto II, LSR II (BD Biosciences, Franklin Lakes, NJ, USA) equipped with a 561 nm laser (PE) [16]. 10,000 cells were analyzed in each treatment group. Imaging flow cytometry was performed using an Amnis ImageStreamX MK I (Cytek Biosciences, Fremont, CA, USA) with a 561 nm laser and a 40× magnification as described before [16]. For imaging flow cytometry 1000 cells of each treatment group have been analyzed.

### Statistical analysis

Statistical analysis was performed via GraphPad Prism V5.01 (GraphPad Software, CA, USA; https://www.graphpad.com). The data were checked for a Gaussian distribution via the Kolmogorov–Smirnov test. All groups were tested for statistically significant differences via two-way ANOVA and the Bonferroni post hoc correction. All values are displayed as the means with standard deviations. *P* values ≤ 0.05 were considered statistically significant.

## Results

### Transfected exosomes have a typical morphology

**Fig. 1:**
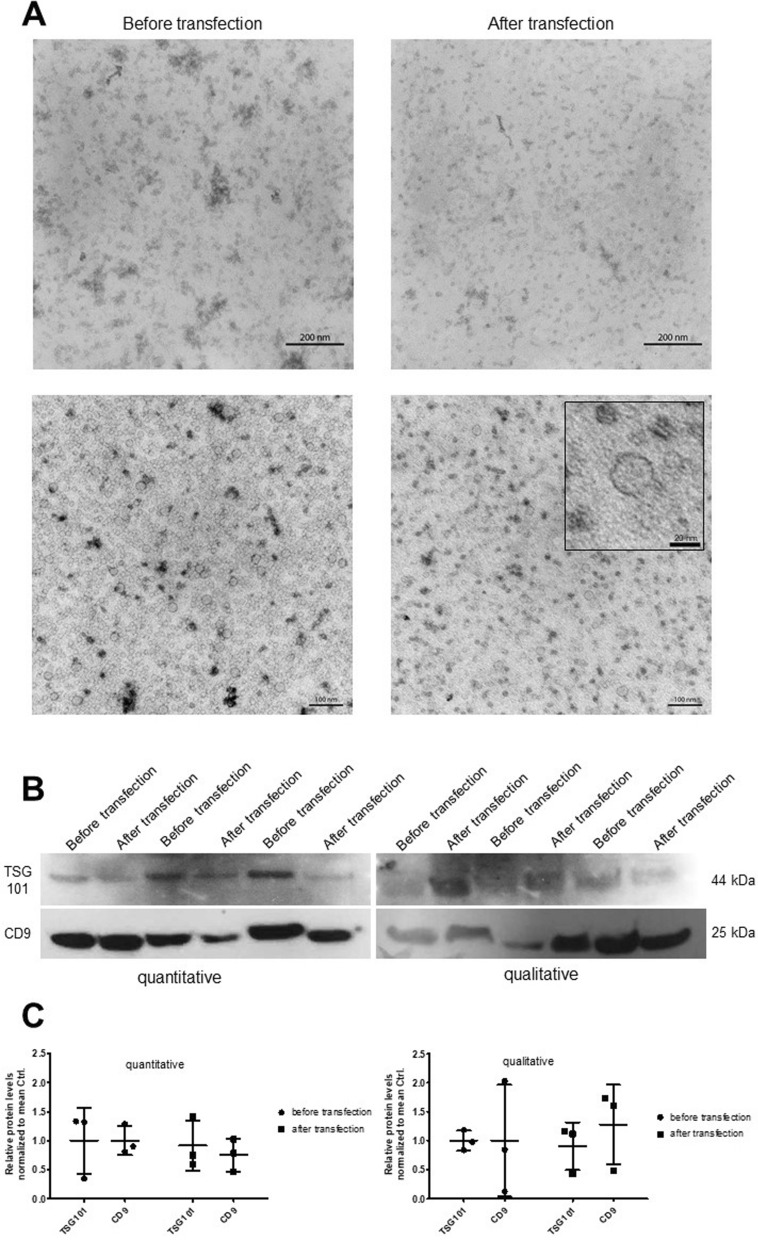
Exosome characterization before and after transfection. **A** Exosomes were isolated with ExoQuick-TC before and after transfection with Cy3-miRCtrl with the Exo-Fect™ siRNA/miRNA Transfection Kit. Exosomes were prepared for TEM. The exosomes showed a typical shape and sizes of 20–30 nm (*box*). There was no difference in exosome number after transfection. Exosomes appeared cleaner and more homogeneously distributed after transfection. Scale bars = 200 nm and 100 nm, box = 20 nm. **B** Exosomes from three independent transfections with Cy3-miRCtrl were precipitated with ExoQuick-TC and analyzed by Western blotting for the exosomal marker proteins TSG101 and CD9 quantitatively and qualitatively. There were no differences in exosomal marker levels between the two analysis approaches. **C** The Western blot bands were analyzed semiquantitatively via Fiji. The data shown are the means ± SDs. There were no significant differences in exosomal marker expression before and after the transfection of exosomes

To characterize the exosomes derived from the cell culture supernatants before and after transfection with the Exo-Fect™ siRNA/miRNA Transfection Kit (System Biosciences), exosome pellets were prepared for TEM analysis. We observed that the exosomes exhibited a characteristic cup-shaped morphology with sizes ranging from 20 to 30 nm. Interestingly, exosomes tended to aggregate and form clusters before transfection, a phenomenon that is absent in transfected exosomes. These exosomes displayed a homogenous distribution and were distinct from each other. However, we did not observe differences in exosome number or size (Fig. 1 A, Suppl. Figure 1).

### Transfected exosomes exhibit typical marker expression

To further characterize the isolated exosomes, we compared them to their host podocytes by Western blots for the specific exosomal marker proteins TSG101 and CD9. Both marker proteins were highly expressed in exosomes and host podocytes. We observed a strong enrichment of CD9 in exosomes compared to host podocytes. In contrast, TSG101 levels were decreased in exosomes compared to host podocytes (Suppl. Figure 2). To compare the exosomal marker proteins TSG101 and CD9 in isolated exosomes before and after transfection, we performed qualitative and quantitative Western blots (Fig. 1B, C). The quantitative Western blots (equal volumes) revealed a slight, nonsignificant reduction in TSG101 and CD9 expression in the transfected exosomes (Fig. 1B, C), indicating a reduction in the number of exosomes after transfection. With respect to the qualitative (equal protein input) Western blots, we detected slightly increased CD9 signals in the exosomes post transfection compared with those in the exosomes prior to transfection, indicating increased exosome purity after transfection.

### Podocyte uptake of exosomes loaded with Cy3-labeled pre-miRNA

**Fig. 2:**
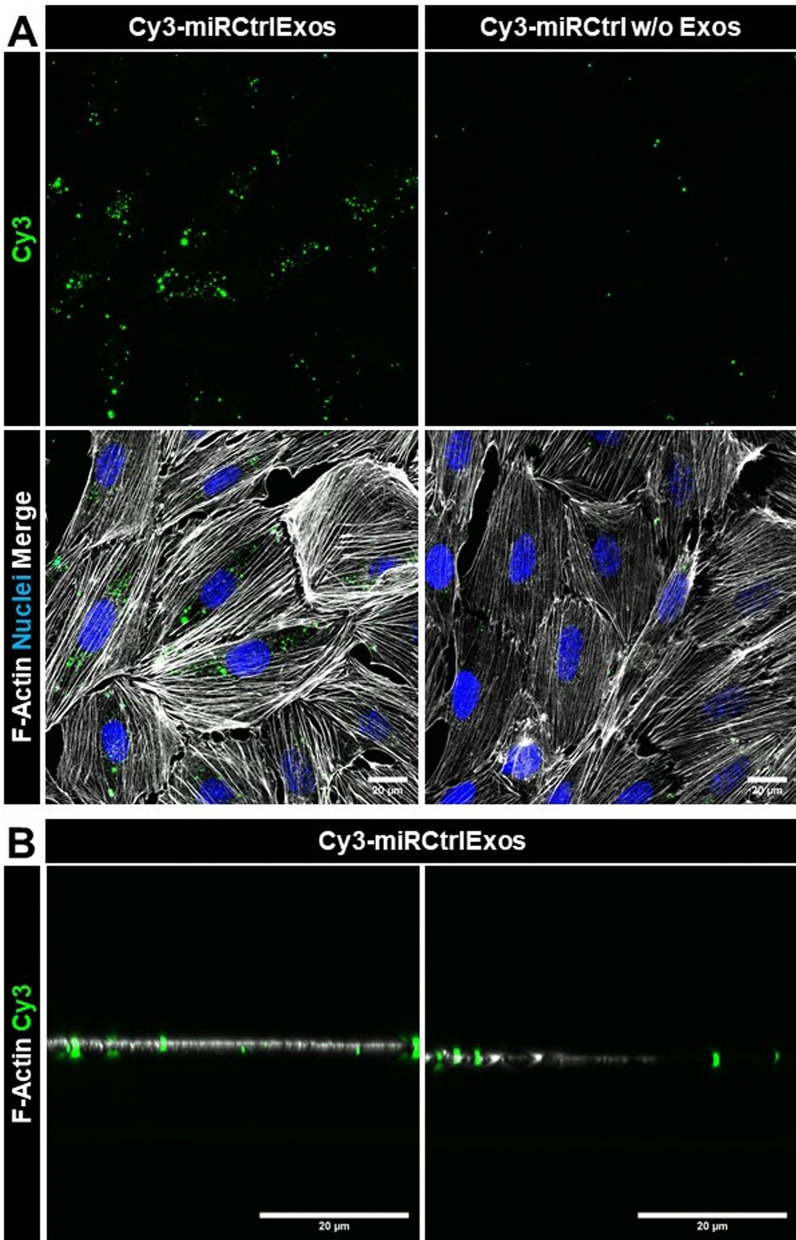
Cultured podocytes internalize cargo from exosomes loaded with Cy3-Ctrl miRNA. **A** Cultured murine podocytes were treated for 48 h with exosomes previously transfected with Cy3-Ctrl-miRNA (Cy3-miRCtrlExos) or with Cy3-Ctrl-miRNA w/o exosomes (Cy3-miRCtrl w/o Exos). Podocytes treated with Cy3-miRCtrl w/o Exos presented only a few Cy3-spots. Podocytes treated with transfected exosomes presented intracellular Cy3-signals, as shown by xyz (**A**) and **B** xzy-projections. Scale bars = 20 µm. F-Actin is stained with phalloidin. Nuclei are stained with DAPI. Maximum intensity projections (MIPs) of z-stacks

We incubated cultured murine podocytes with Cy3-labeled control miRNA-loaded exosomes (Cy3-miRCtrlExos) for 48 h. To rule out any background staining from unloaded Cy3-fluorophores, we incubated the podocytes with Cy3-labeled Ctrl-miRNA without exosomes (Cy3-miRCtrl w/o Exos) that had undergone the same washing procedure as the transfected exosomes, followed by phalloidin and DAPI staining. We observed the internalization of Cy3-miRCtrlExos by podocytes via cLSM (Fig. 2A). This was additionally confirmed by cLSM in the xzy-plane (Fig. 2B). Since the Cy3-signals were found in close proximity to F-actin filaments, we assumed that the exosomal miRNA cargo was taken up by the cultured podocytes. In contrast, Cy3-signals were rarely detected in samples treated with only Cy3-miRCtrl w/o Exos. These results indicate the selectivity of the exosome transfection procedure and suggest that unloaded Cy3-miRNA signals do not play a significant role in this context.

**Fig. 3:**
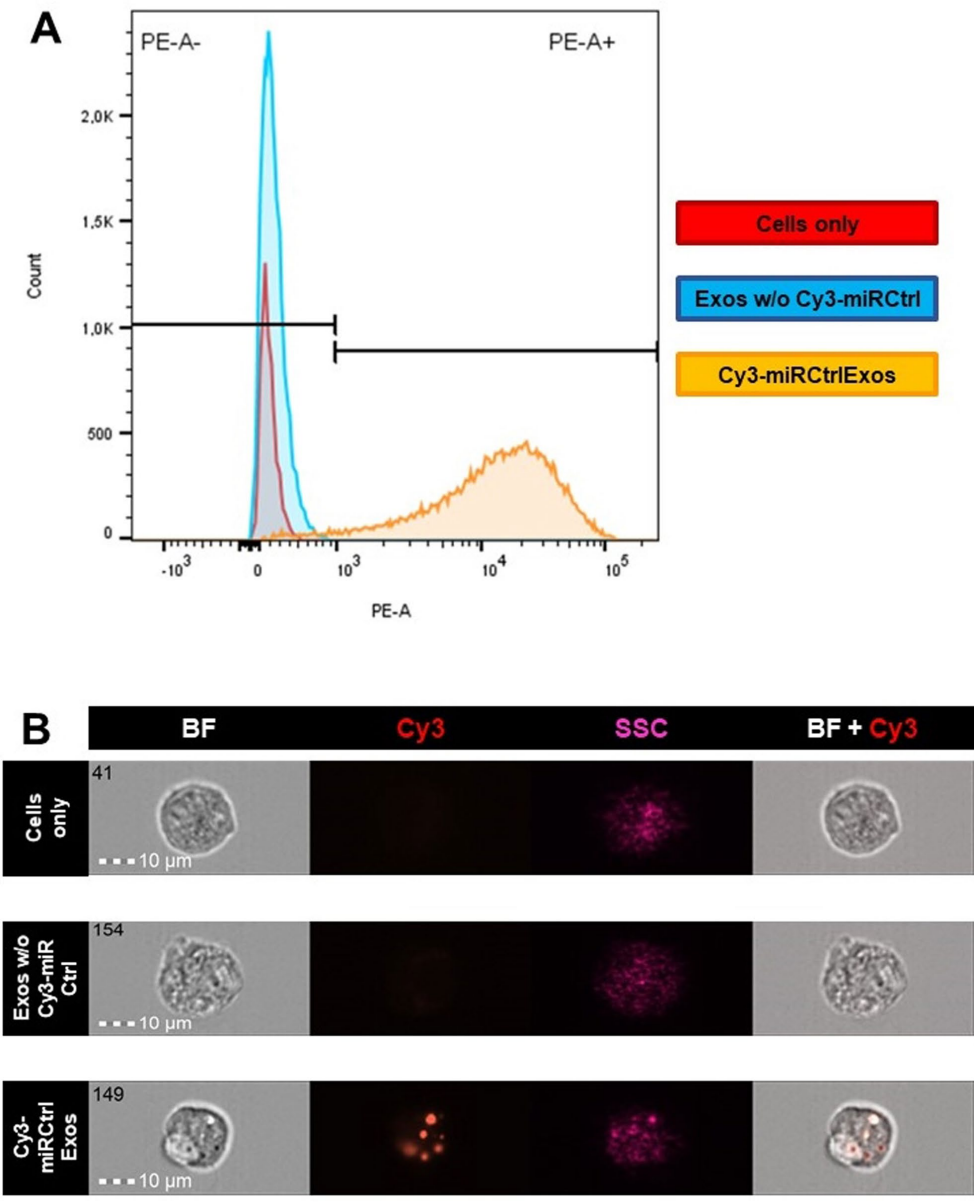
Flow cytometry and imaging flow cytometry show a high internalization efficiency. **A** After treatment with Cy3-miRCtrlExos, 96.5% of all podocytes were positive for Cy3 (*orange line*, PE-A channel). Podocyte treated with Exos w/o Cy3-miRCtrl showed only 0.15% Cy3-positive podocytes (*blue line*, PE-A channel). Untreated podocytes were used as the reference (*red line*, PE-channel). **B** Imaging flow cytometry revealed Cy3-positive signals (*red*) only in podocytes that were treated with Cy3-miRCtrlExos. No signals were observed in the other two treatment groups. In brightfield (BF) and side scatter (SSC, purple), no morphological differences between the groups were found. Scale bars = 10 µm

To assess the efficient uptake of exosomal cargo, we performed flow cytometry and imaging flow cytometry of treated podocytes. As revealed by flow cytometry, we could observe an uptake efficiency of 96.5% in podocytes treated with Cy3-miRCtrlExos whereas podocytes treated with Exos w/o Cy3-miRCtrl only show 0.15% efficiency (Fig. 3A; Suppl. Figure 3). A similar pattern was observed using imaging flow cytometry. We observed an uptake efficiency of 86.8% in podocytes treated with Cy3-miRCtrl Exos-treated and 1.38% in those treated with Exos w/o Cy3-miRCtrl (Fig. 3C; Suppl. Figure 3). Additionally, Cy3-positive signals were found in Cy3-miRCtrExos-treated podocytes, whereas the other treatment groups remained Cy3-negative. No morphological differences were observed between the individual treatment groups (Fig. 3C; Suppl. Figure 3).

### Internalization of the exosomal cargo was confirmed by co-staining for CD9, Rab5 and Rab7

**Fig. 4:**
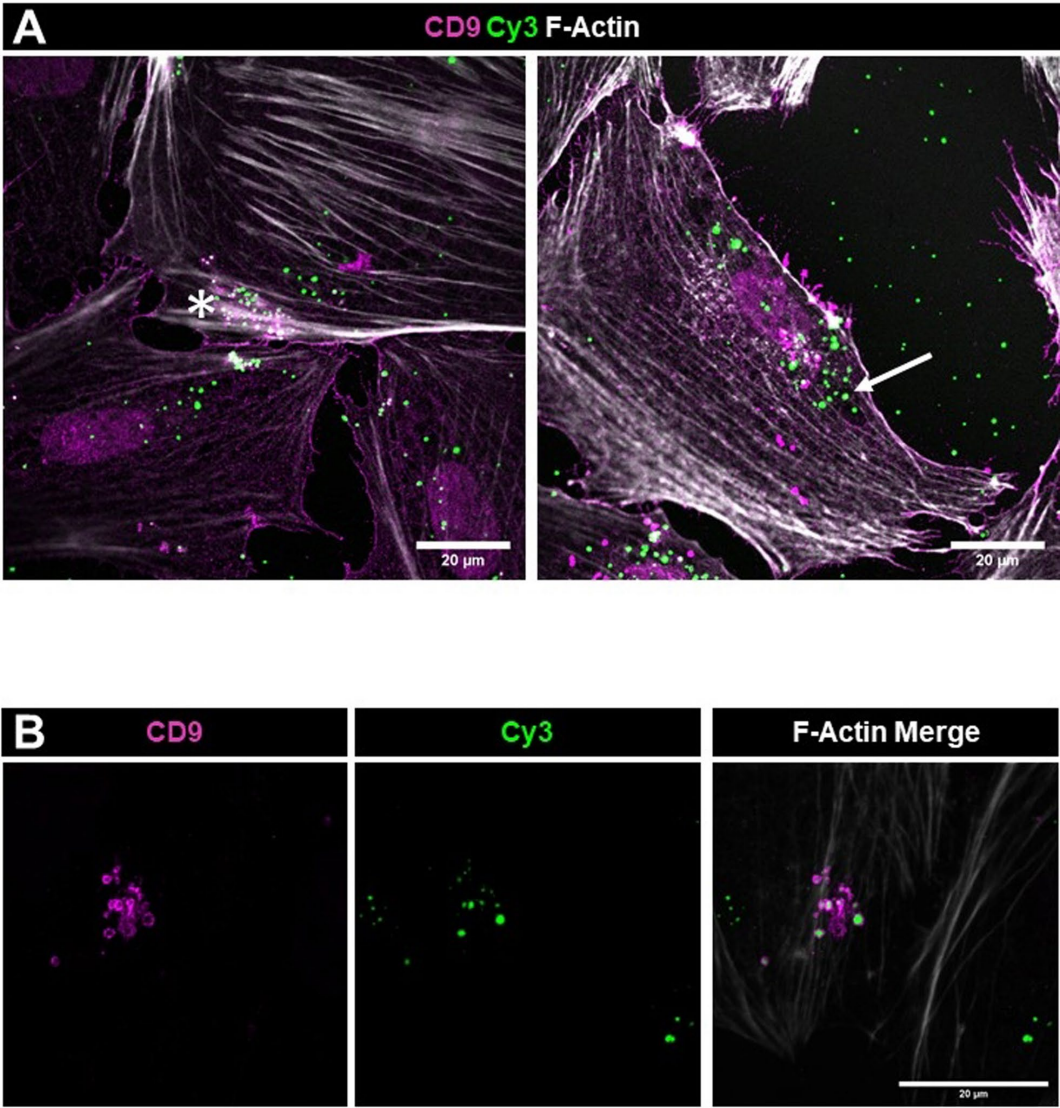
Staining for CD9 confirms the internalization of the exosomal cargo. **A** After treatment with Cy3-miRCtrlExos, podocytes were stained with an antibody against CD9. Podocytes presented Cy3-signals inside the cell body. The Cy3-signals were either CD9-negative (**A**, *arrow*) or enclosed in CD9-positive vesicles (**A**, *asterisk* and **B**). F-Actin is stained with phalloidin. Scale bars = 20 µm. Single planes (**A**) and MIP of z-stacks (**B**)

To further investigate the internalization mechanisms of Cy3-miRCtrlExos, we stained podocytes with an antibody against CD9, a membrane and exosomal marker protein. Our analysis showed that Cy3-positive signals were predominantly localized within the podocyte cell bodies (Fig. 4A), confirming an efficient uptake of the exosomes. Two different types of exosomes were detected: Exosomes with CD9-negative Cy3-spots (Fig. 4A, arrow), as observed after membrane fusion and exosomes with CD9-positive Cy3-spots (Fig. 4A, asterisk; Fig. 4B), that are characteristic for an endocytotic uptake mechanism. The observed vesicles have had a diameter between 200 and 700 nm.

**Fig. 5:**
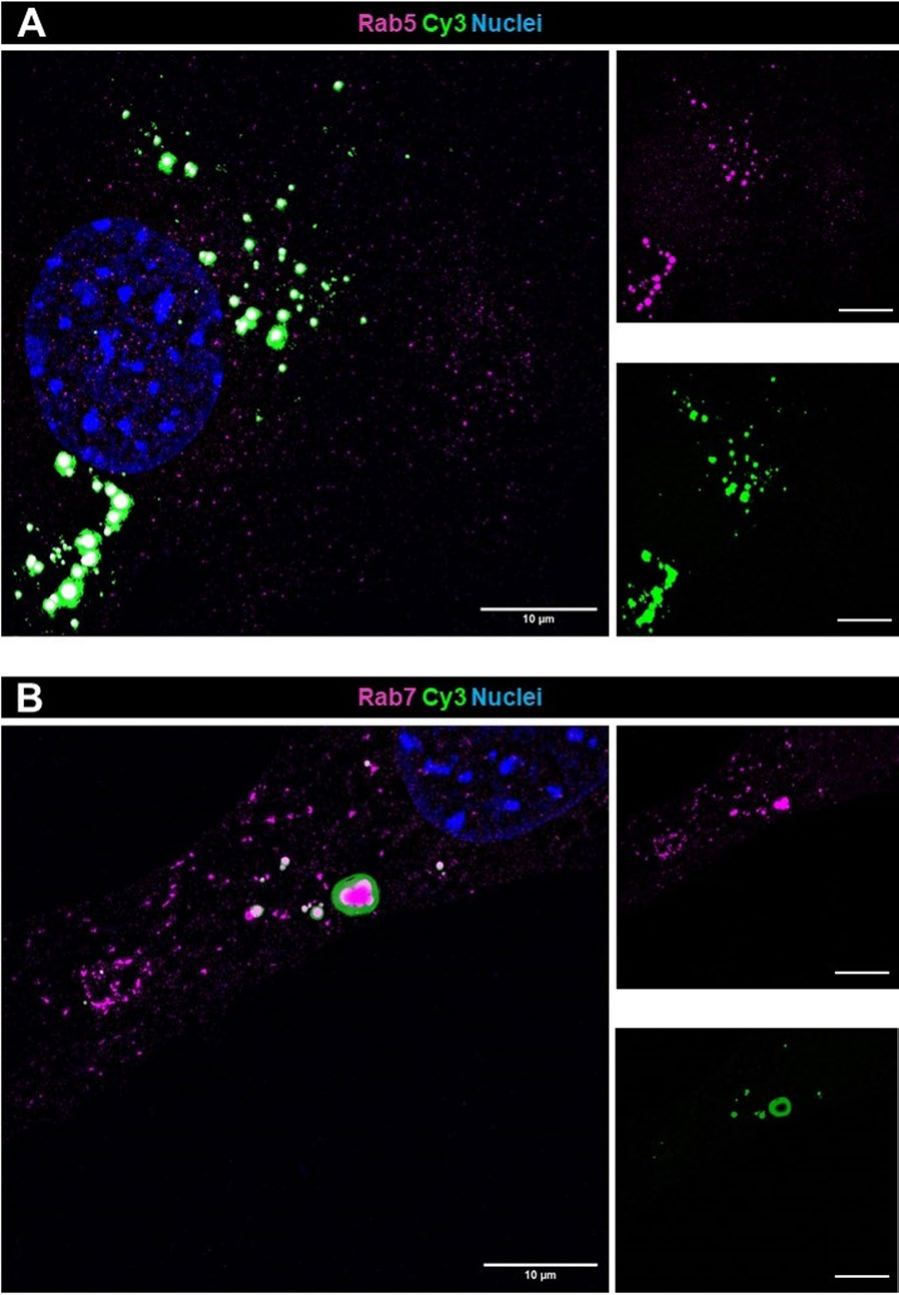
Staining for the early endosomal marker Rab5 and the lysosomal marker Rab7 reveals endocytotic exosome cargo uptake rather than lysosomal degradation. Cultured podocytes were stained with antibodies against Rab5 (**A**) and Rab7 (**B**) after treatment with Cy3-miRCtrlExos. **A** Podocytes widely colocalized with Cy3 and the early endosomal marker Rab5. **B** Cy3-positive signals rarely colocalized with the lysosomal marker Rab7. To visualize small particles, the microscope settings were set to signal saturation. Nuclei are stained with DAPI. Scale bars = 10 µm, 20 µm in single channels. MIPs of z-stacks

To further elucidate the mechanism of exosomal cargo uptake, we stained our cells with an antibody against the early endosome marker Rab5. By using superresolution SIM, we observed broad colocalization of Cy3 and Rab5 (Fig. 5A). Compared with that of Rab5, the colocalization of Rab7, a late endosome and lysosome marker protein, with Cy3-signals was lower (Fig. 5B).

### Time and dose dependence of exosomal cargo internalization

**Fig. 6:**
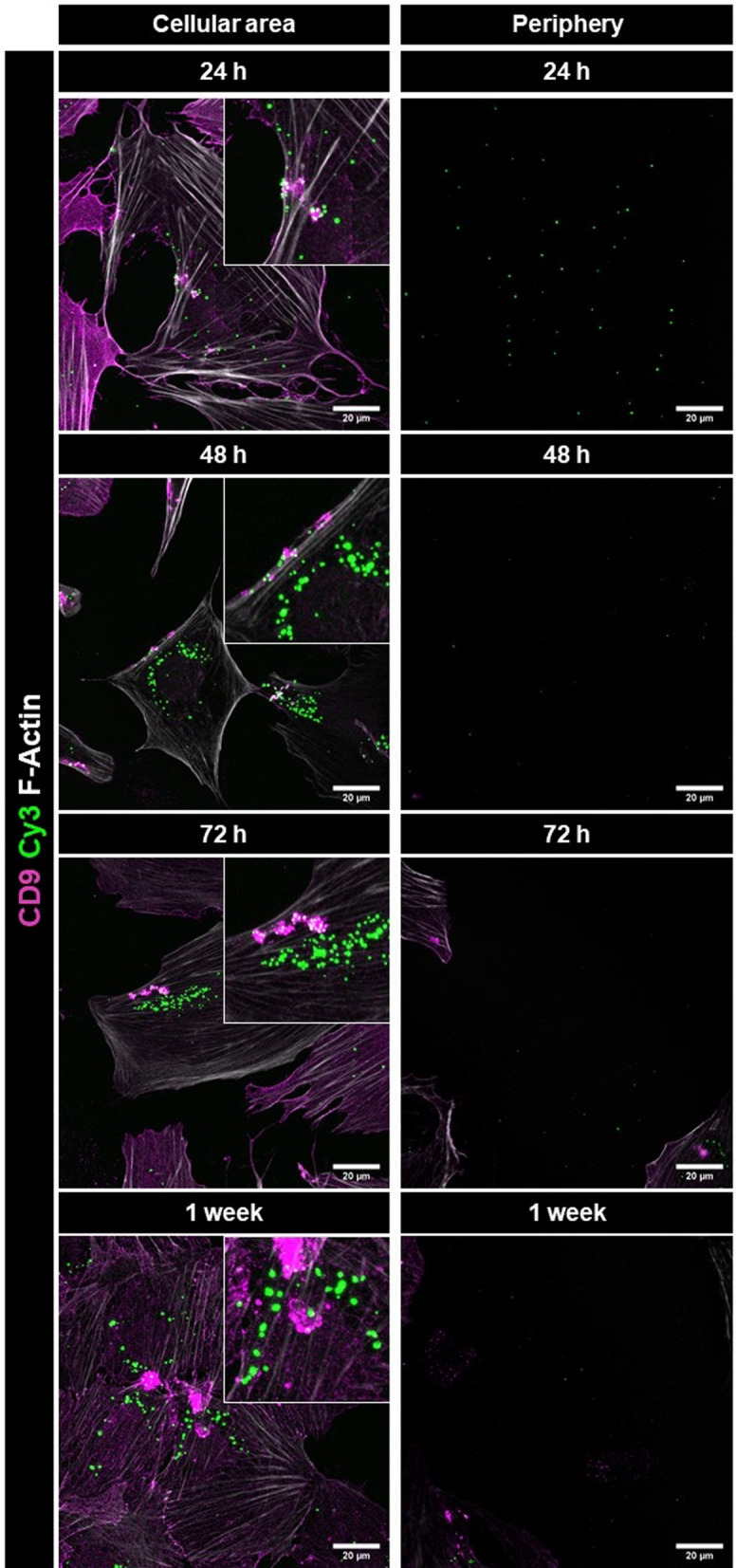
Time dependence of exosomal cargo internalization. Podocytes were treated with Cy3-miRCtrlExos for 24, 48, 72 h and 1 week, followed by staining for CD9. Immunofluorescence micrographs were acquired from the cellular area as well as from the cell-free periphery. The number of Cy3-spots decreased in the periphery and increased within the cells over time. F-Actin is stained with phalloidin. Scale bars = 20 µm. MIPs of z-stacks (cellular area) and single planes (periphery)

To investigate the dynamics of exosomal cargo internalization, we treated podocytes with Cy3-miRCtrlExos for 4 h, 12 h, 24 h, 48 h and 1 week. We observed that Cy3-Ctrl signals were internalized by the cells as early as after 4 h of treatment. Cy3-Ctrl signals could be seen within the cells as well as in the cell-free periphery in equal amounts at time-points up to 24 h of treatment (Fig. 6; Suppl. Figure 4 B). We identified Cy3-miRCtrl signals extracellularly as well as internalized by podocytes. Additionally, we observed an accumulation of Cy3-Ctrl signals in close proximity to the cell borders, as shown at higher magnification in Fig. 5. After 48 h, only a few Cy3-Ctrl signals were visible in the periphery, whereas Cy3-Ctrl signals accumulated in the cellular area (Fig. 6; Suppl. Figure 4B). Over a period of one week, the Cy3-Ctrl signals further disappeared from the cell-free areas, and all the detected Cy3-Ctrl signals were localized within the cells and partially accumulated in CD9-positive vesicles (Fig. 6). Furthermore, we treated the cells with different exosome concentrations (18.75, 37.5, 75 and 150 µg per well) to investigate dose-dependent effects. A clear correlation was observed between exosome dosage and exosomal cargo uptake by podocytes (Suppl. Figure 4 A).

### Treatment of podocytes with pre-miR-21-loaded exosomes leads to the overexpression of mature miR-21

**Fig. 7:**
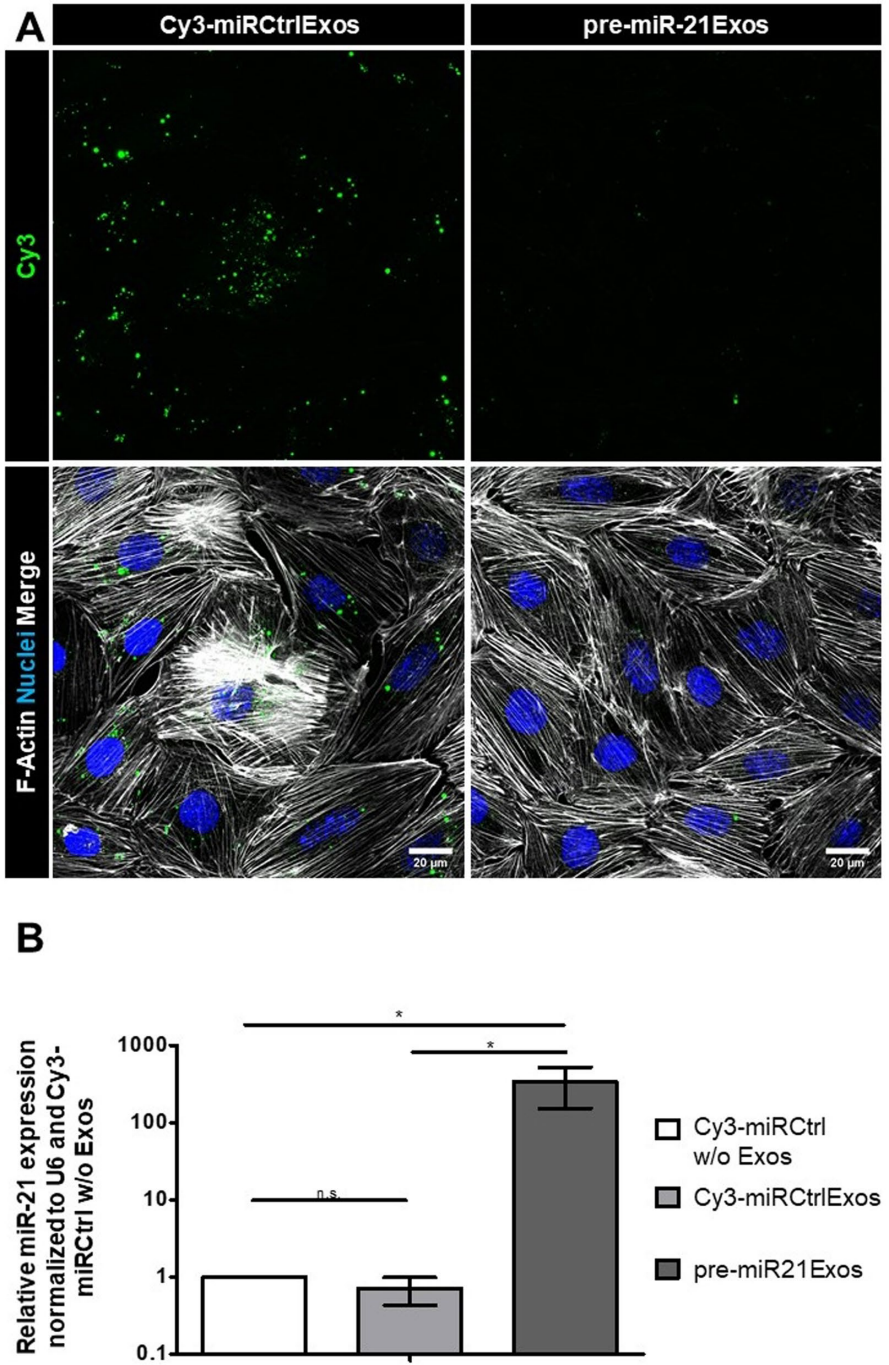
Exosomes loaded with pre-miR-21 upregulate miR-21 expression in podocytes. **A** Podocytes were treated with Cy3-miRCtrlExos and pre-miR-21Exos for 48 h. Podocytes internalized Cy3-Ctrl, whereas almost no Cy3-fluorescence was detected in unlabeled pre-miR-21Exos-treated samples. F-Actin is stained with phalloidin. Nuclei are stained with DAPI. Scale bars = 20 µm. MIPs of z-stacks. **B** Treatment with pre-miR-21Exos led to a 338-fold upregulation of miR-21 in podocytes (*p* = 0.03). The graph shows the relative miR-21 expression normalized to U6 and Cy3-miRCtrl w/o Exos as the mean ± SD

After successful exosomal cargo tracking in podocytes, we investigated whether exosomes could serve as delivery vehicles for pre-miRNAs. Therefore, we treated cultured podocytes with exosomes transfected with pre-miR-21 (pre-miR-21Exos) and Cy3-miRCtrl (Cy3-miRCtrlExos). We analyzed the internalization efficiency by cLSM. To evaluate whether the loaded pre-miR-21 was processed to its mature form, we isolated total RNA from treated podocytes and measured the amount of mature miR-21 via RT‒qPCR. Prior to this, we performed several washing steps to eliminate uninternalized exosomes. We detected Cy3-signals within all podocytes treated with Cy3-miRCtrlExos, indicating full internalization of the exosomal cargo (Fig. 7A). Podocytes treated with unlabeled pre-miR-21Exos were free of Cy3-fluorescence. RT‒qPCR revealed a significant 338-fold (*p* = 0.03) increase in mature miR-21 compared with that in Cy3-miRCtrl w/o Exos-treated podocytes. In contrast, podocytes treated with Cy3-miRCtrlExos showed no significant difference in mature miR-21 expression levels (0.71-fold, *p* = 0.15) compared with those in Cy3-miRCtrl w/o Exos Ctrl. However, these samples differed significantly from the pre-miR-21Exos-treated samples (*p* = 0.03) (Fig. 7B).

### Treatment of podocytes with filamin A siRNA-loaded exosomes leads to the downregulation of filamin A protein expression

**Fig. 8:**
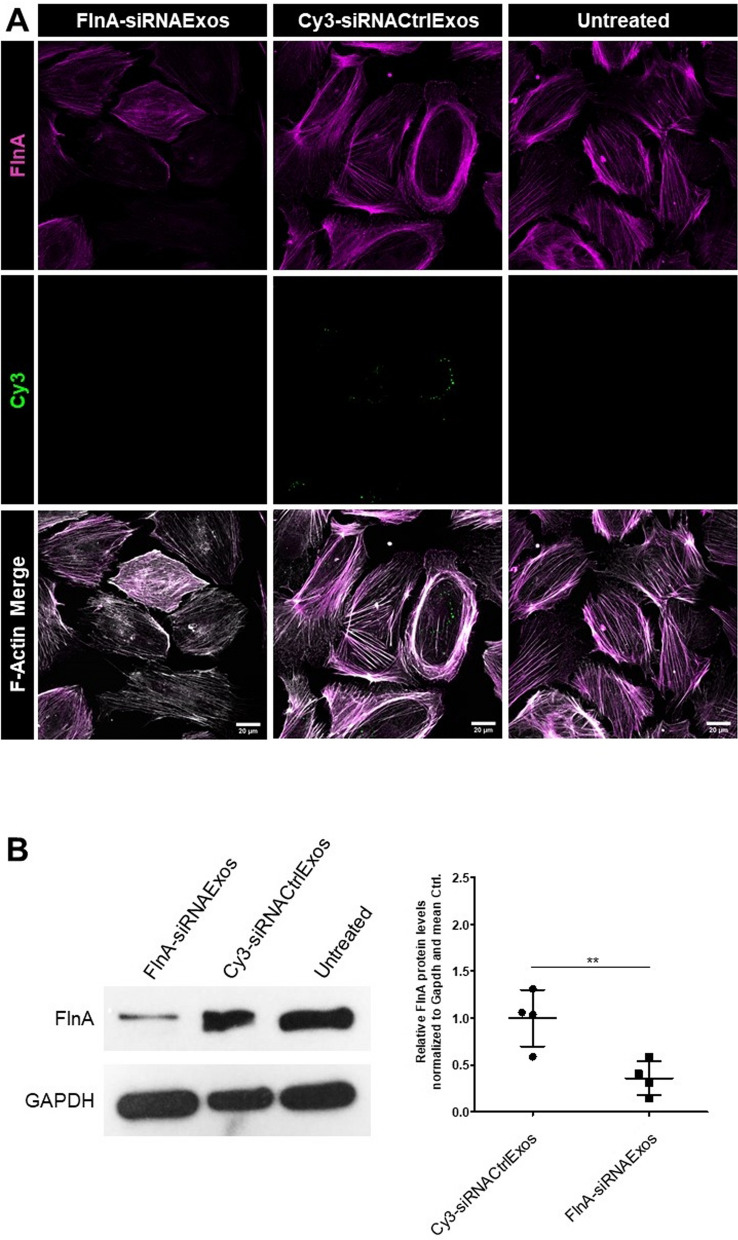
Exosomes loaded with FlnA-siRNA downregulate FlnA expression in podocytes. **A** Podocytes were treated with Cy3-siRNACtrlExos or FlnA-siRNAExos for 48 h. Podocytes internalized Cy3-siRNACtrl, whereas no Cy3-signals were detected in the untreated and FlnA-siRNAExo samples. FlnA fluorescence decreased in the FlnA-siRNA Exo-treated samples. F-Actin was stained with phalloidin. Scale bars = 20 µm. MIPs of z-stacks. **B** Treatment of podocytes with FlnA-siRNAExos resulted in the downregulation of FlnA expression to 0.36 (*p* = 0.006), as shown by Western blot analysis

In addition to pre-miRs as cargo molecules, we investigated whether siRNAs could also be successfully transferred to cultured podocytes via exosome transfection. For this purpose, we used a well-established siRNA against filamin A (FlnA) mRNA as a representative target (FlnA-siRNAExos). Furthermore, we used Cy3-labeled, scrambled siRNA-Ctrl as an internalization control (Cy3-siRNACtrlExos). In the comparative analysis shown in Fig. 7A, podocytes treated with Cy3-siRNACtrlExos presented fewer and weaker Cy3-signals than cells treated with Cy3-miRCtrlExos (Fig. 6A). Notably, podocytes treated with FlnA-siRNAExos completely lacked Cy3-signals, which was very similar to the results of the untreated control (Fig. 8A). This finding indicates a very reliable exosome clean-up. Compared with Cy3-siRNACtrlExo-treated and untreated cells, FlnA expression was downregulated in cells incubated with FlnA-siRNAExos (Fig. 8A), whereas no change in FlnA expression was detected in the controls. Approximately 50% of the treated podocytes per field of view showed almost no FlnA protein staining, whereas all control cells were FlnA-positive. These results were confirmed by Western blot analysis, which revealed a significant (*p* = 0.006) decrease in FlnA expression to 0.36 in the samples treated with FlnA-siRNAExos compared with those in the Cy3-siRNACtrlExos and untreated controls (Fig. 8B).

To further validate these results, a well-established siRNA targeting the mRNA of the focal adhesion protein paxillin (Pxn) was used. Podocytes treated with Pxn-siRNAExos exhibited a pronounced downregulation of Pxn in nearly all cells, as demonstrated by immunofluorescence staining (Suppl. Figure 5).

## Discussion

In the past, exosomes have been shown to be effective carriers for the transfer of small RNAs to different cell types, making them promising therapeutic tools for the treatment of various diseases [7, 17–19]. However, in podocyte diseases, which account for 80% of all CKD cases, there are no treatment options based on specific target RNAs loaded into exosomes. However, this new approach could close the large gap in the currently limited treatment options.

The present exosome-based studies mostly used indirect loading strategies. This includes exosomes isolated from special cell types that naturally overexpress RNAs, such as stem cells. In their study, Jin et al. treated diabetic mice with miR-486-5p-enriched exosomes secreted by adipose-derived stem cells, which improved podocyte damage by regulating the Smad1/mTOR signaling pathway [8]. Another common indirect approach is the transfection of donor cells with specific small RNAs and the subsequent isolation of enriched exosomes secreted by these cells. Zhang et al. transfected HEK293 cells with miR-145-5p, isolated miR-145-5p-enriched exosomes and then co-cultured these exosomes with podocytes. Therefore, they observed toxic effects of the miR-145-5p-enriched vesicles on cultured podocytes [8, 9]. However, both strategies have considerable disadvantages. First, they lack specificity, as many other endogenous exosomal small RNAs are present in addition to the target RNAs. Second, the efficiency of packaging transfected small RNAs into exosomes within donor cells is often insufficient, resulting in lower transfection rates and poor reproducibility. Third, transfection reagents are difficult to remove completely from isolated exosomes and can therefore be cytotoxic to recipient cells [10].

A promising alternative approach is the direct transfection of exosomes, which are already being used in cancer research [20]. However, it is still unclear whether directly transfected exosomes can effectively deliver small RNAs into podocytes. The aim of this study was to directly load exosomes isolated from cultured mouse podocytes with small RNAs, which were then incubated with podocytes.

The isolated exosomes presented the classical cup shape and size of 20–30 nm before and after transfection. This relatively small exosome size is typical for podocyte-derived exosomes and has been published previously [21, 22]. Furthermore, there was no significant decrease in the quantity or quality of exosomes following the transfection of the prepared exosomes. Interestingly, we noted a more homogenous exosome distribution after transfection via TEM.

To evaluate the efficiency of this method, we used fluorescently-labeled miRNAs and siRNAs to track the uptake of exosomal cargo by podocytes. Currently, most studies use staining protocols to visualize exosome uptake via fluorescent labeling of exosomal membrane components via either immunofluorescence or organic dyes [23]. These methods have various disadvantages since organic dyes are predisposed to stain cellular components other than exosomes and thereby generate false positive results. Immunofluorescence staining using specific antibodies for exosome membrane proteins is very costly and labor intensive and includes various washing steps, which drastically reduce the exosome yield [23]. In the present study, we circumvented these drawbacks by using prelabeled exosomal cargo instead of staining exosomal structural components. This technique not only increases the potential for tracking small RNA transport but also offers significant advantages for tracking exosomes and exosomal cargo. By using this method, we were able to show that cultured podocytes internalize the fluorescentlylabeled small RNAs delivered by directly transfected exosomes.

To reveal the mechanism of uptake, we performed additional experiments. In general, exosomes can be internalized in different ways, such as clathrin-dependent [24, 25] and caveolae-dependent [25, 26] endocytosis [25, 27] and macro- and pinocytosis [24, 25] or receptor-mediated endocytosis. We observed that the internalized small RNAs are localized either within the cytosol or encapsulated in CD9-positive vesicles. To determine the dominant vesicle type, we stained for Rab5, a marker for early endosomes [28], and Rab7, a marker for late endosomes [29]. After staining podocytes with specific antibodies, we found that most Cy3-fluorescence colocalized with Rab5 and, to a lesser extent, with Rab7. These findings suggest that the uptake of exosomal cargo by podocytes in vitro occurs via endocytosis. However, the exact mechanisms of exosomal cargo uptake by podocytes remain rather unclear and require further studies.

To determine whether the transfected small RNAs remained functional, exosomes were loaded with specific siRNAs against FlnA and Pxn as representative targets since they are essential for podocyte cytoskeleton integrity and highly expressed in cultured podocytes [30, 31]. We found that FlnA and Pxn were almost completely downregulated in podocytes that were treated with FlnA/Pxn-siRNA exosomes confirming that the siRNAs remained functionally active.

A similar result was also observed for pre-miR-21-loaded exosomes. MiR-21 has been shown to be upregulated in kidney injury and in the urinary exosomes of CKD patients [14]. We found that mature miR-21 was strongly upregulated in podocytes after pre-miR-21-exosome treatment.

During the biogenesis of miRNAs, they undergo several processing steps. After transcription, miRNAs are present as pri-miRs with a length of >1 kb and are further processed intranuclearly into 60–70 nt long pre-miRs with a stem‒loop structure. These pre-miRs are exported into the cytosol and then cleaved by Dicers into mature miRNA and an opposing strand, which is further degraded [32]. Using Taqman probes that are specific for mature miR-21 and can distinguish between pre-miRs and their mature forms with 2000-fold higher efficiency [33], we observed that the transfected pre-miR-21 was processed into mature miR-21 by the recipient podocytes, confirming their integrity. These results are consistent with previous findings that RNAs packed in exosomes are protected from degradation by RNases [34].

Our findings may open up great possibilities for various experimental approaches in podocyte research. The uptake of exosomes by podocytes has been reported several times before [35, 36], but to the best of our knowledge, this is the first study to describe small RNA uptake in podocytes by directly transfected exosomes.

Podocytes, a postmitotic cell type, undergo cell cycle arrest even under most in vitro conditions [37]. Like other postmitotic cells, podocytes are difficult to transfect and highly sensitive to transfection reagents [37]. Here, we show that directly transfected exosomes could represent a promising alternative to overcome these challenges. Direct exosome transfection could also play a promising role in the targeted, therapeutic administration of small RNAs to podocytes in higher organisms. Evidence from oncology research suggests that cells are more prone to phagocytizing exosomes than liposomes, for example, which are currently the gold standard in RNA delivery trials [38]. Moreover, exosomes are less immunogenic than liposomes and express damage-specific surface markers that facilitate specific uptake by injured cells [39]. Taken together, directly transfected exosomes represent a promising clinical strategy for treating podocyte-related diseases since they allow the targeted delivery of miRNA or siRNA. This approach can regulate key molecular pathways, reduce inflammation, prevent apoptosis, restore cytoskeletal integrity, and possibly mitigate proteinuria, thereby preserving renal function.

## Conclusions

In summary, we have shown that direct exosome loading with fluorescently-labeled small RNAs is suitable for exosome tracking approaches in podocytes in vitro. This study is the first to demonstrate the potential of directly transfected exosomes for delivering small RNAs to podocytes in vitro*,* providing an initial indication that exosomes could serve as small RNA carriers for therapeutic strategies in more complex experimental settings.

## Supplementary Information

Additional file 1

Additional file 2

Additional file 3

Additional file 4

Additional file 5

Additional file 6

## Data Availability

The datasets used and/or analyzed during the current study are available from the corresponding author on reasonable request.
