# Supplementary figures and images for: Efficient delivery of small RNAs to podocytes in vitro by direct exosome transfection
Source: J Nanobiotechnology. 2025 May 23;23:373. doi: 10.1186/s12951-025-03426-7 (PMC12100849; doi:10.1186/s12951-025-03426-7)

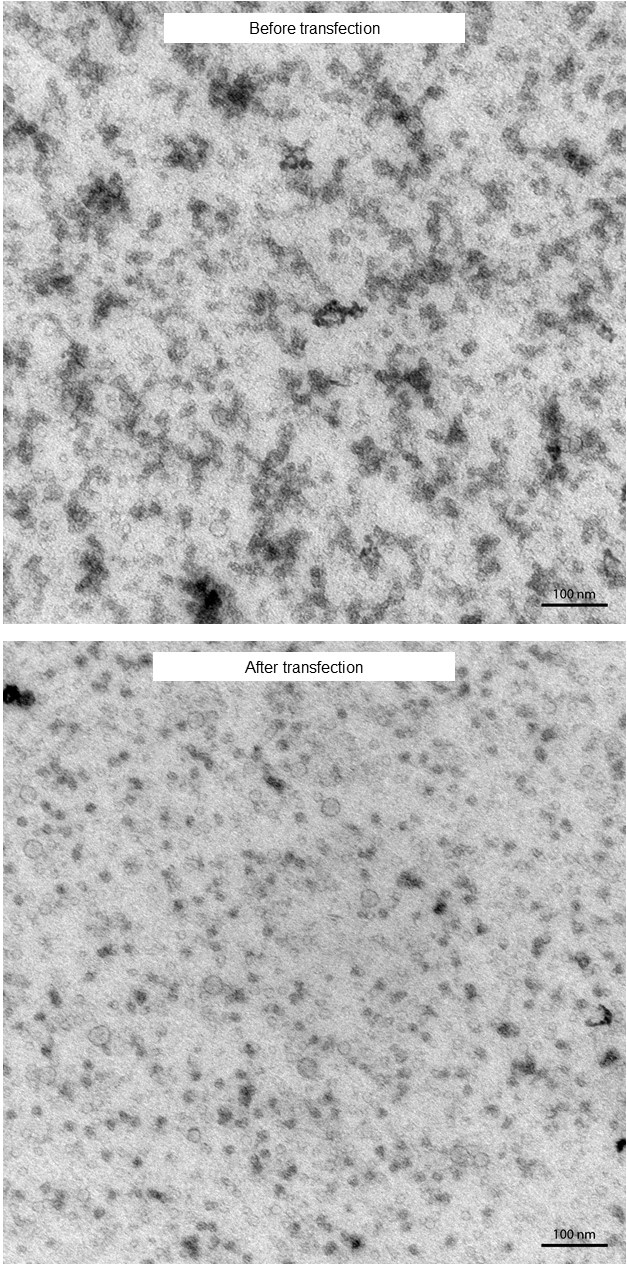

Supplement: Supplementary file 1 — Additional file 1 [file 12951_2025_3426_MOESM1_ESM.jpg]

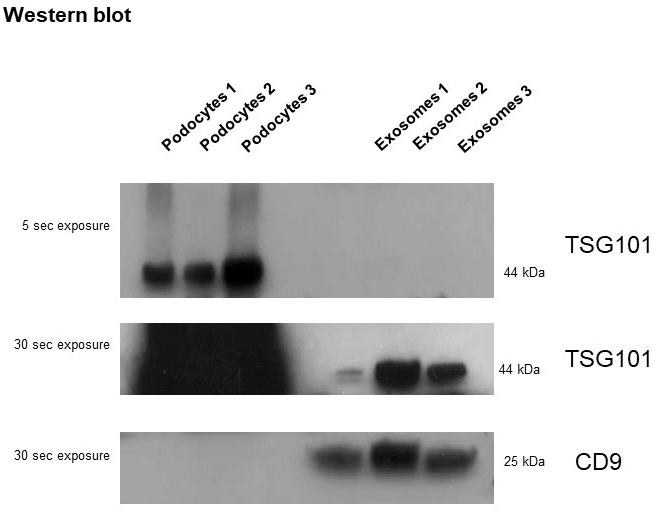

Supplement: Supplementary file 2 — Additional file 2 [file 12951_2025_3426_MOESM2_ESM.jpg]

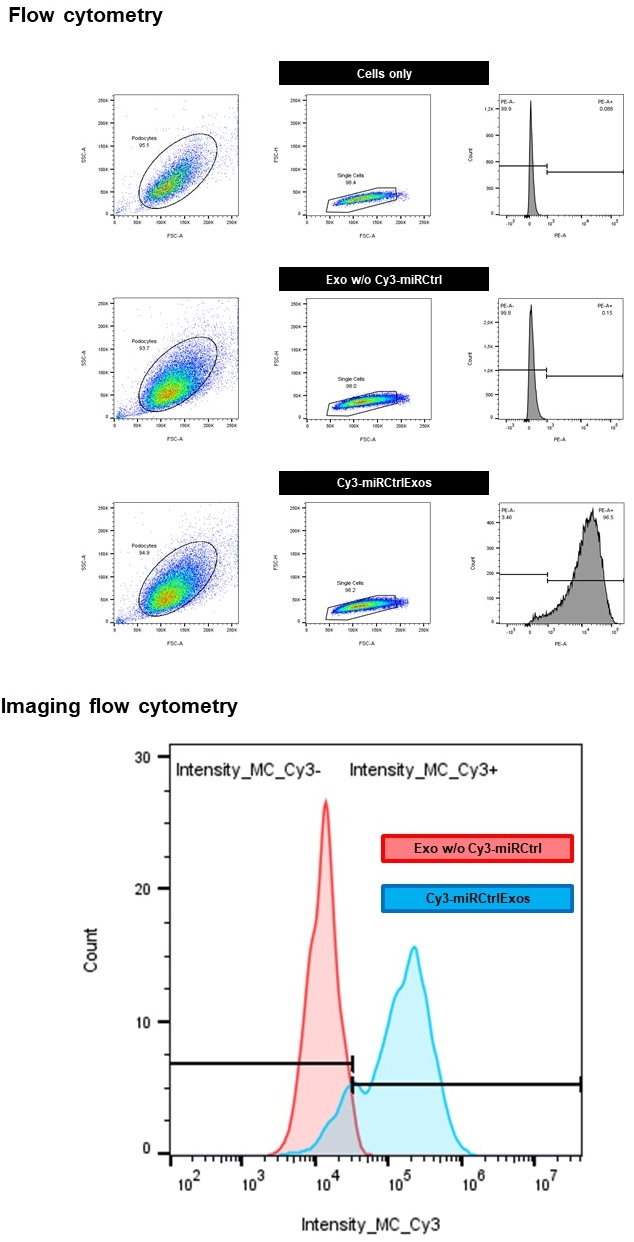

Supplement: Supplementary file 3 — Additional file 3 [file 12951_2025_3426_MOESM3_ESM.jpg]

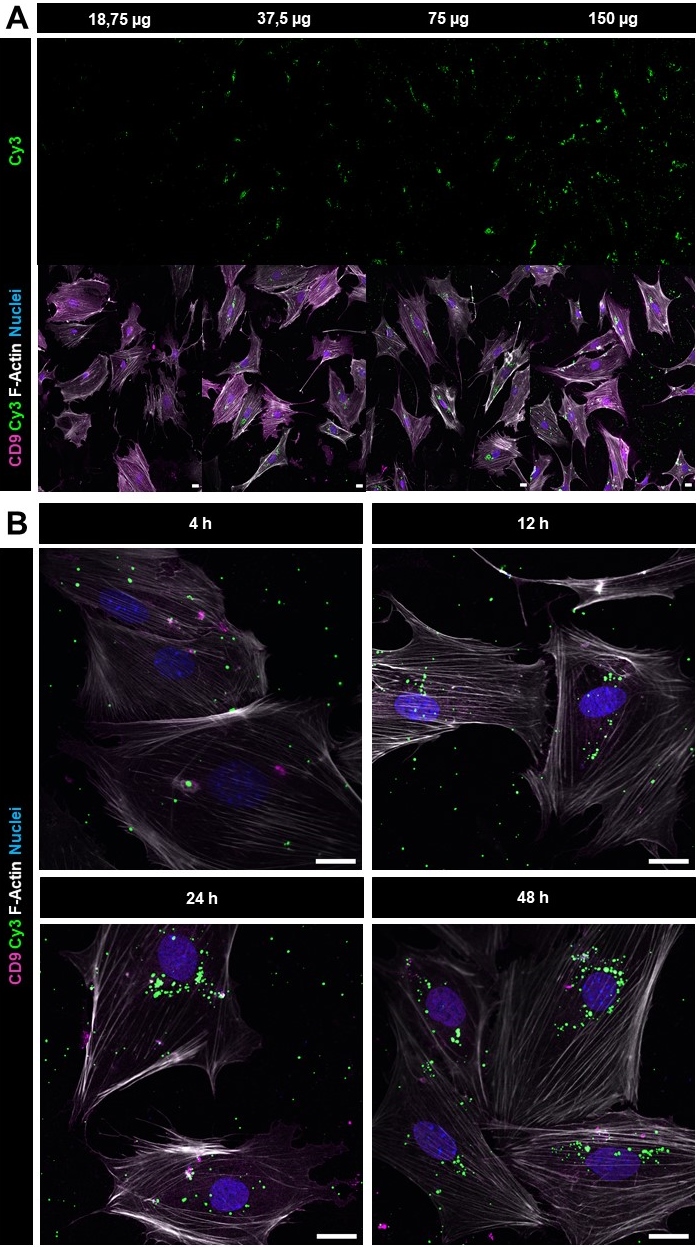

Supplement: Supplementary file 4 — Additional file 4 [file 12951_2025_3426_MOESM4_ESM.jpg]

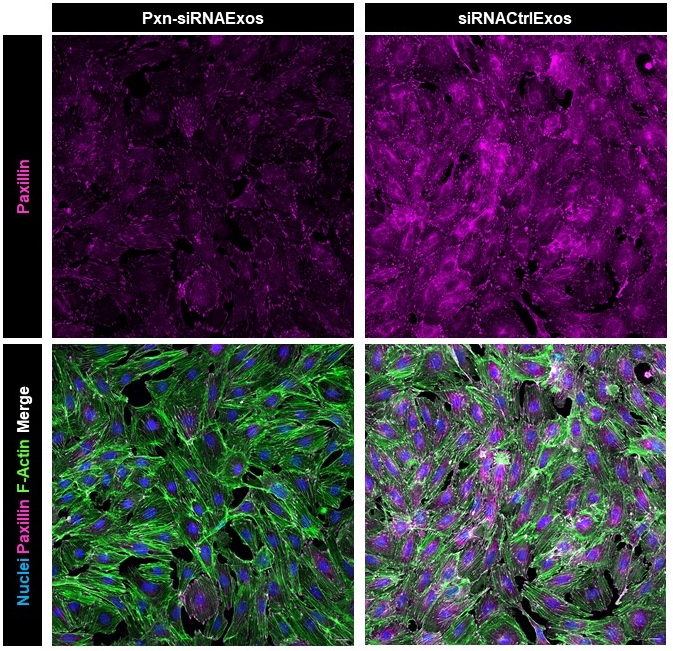

Supplement: Supplementary file 5 — Additional file 5 [file 12951_2025_3426_MOESM5_ESM.jpg]
