# Supplementary material for: Efficient delivery of small RNAs to podocytes in vitro by direct exosome transfection
Source: J Nanobiotechnology. 2025 May 23;23:373. doi: 10.1186/s12951-025-03426-7 (PMC12100849; doi:10.1186/s12951-025-03426-7)
Supplement: Supplementary file 6 — Additional file 6 [file 12951_2025_3426_MOESM6_ESM.pdf]

Supplementary Figure Captions

Supplementary Figure 1: TEM images of exosomes prior and after transfection. Isolated exosomes before and after transfection with Cy3-miRCtrl at 100000 X magnification. The exosomes show a typical cup-shape and size (20-30 nm). Scale bar = 100 nm.

Supplementary Figure 2: Comparison of exosomal marker proteins in podocytes and isolated exosomes. Three independent batches of podocytes and exosomes were precipitated with ExoQuick-TC and analyzed by Western blotting for the exosomal marker proteins TSG101 and CD9. CD9 was highly enriched in exosome samples whereas TSG101 is expressed in both sample types.

Supplementary Figure 3: Flow cytometry and imaging flow cytometry. (A) Normal flow cytometry reveals no differences in cell distribution in terms of size (FSC) and shape (SCC) between the different treatment groups. After treatment with Cy3-miRCtrlExos, 96.5% of all podocytes were positive for Cy3 (PE-A channel). Treated with Exos w/o Cy3-miRCtrl resulted in only 0.15% Cy3-positive podocytes (PE-A channel). Untreated podocytes were used as reference (PE-channel). (B) Imaging flow cytometry reveals Cy3-positive signals (blue line) only in podocytes treated with Cy3-miRCtrlExos. No Cy3-positive signals were found in the samples treated with exosomes without Cy3-miRCtrl (red line).

Supplementary Figure 4: Dose- and time dependence of exosomal cargo internalization. (A) Podocytes were treated with 18.75 µg, 37.5 µg, 75 µg and 150 µg Cy3-miRCtrlExos for 48 hours and stained for CD9. The exosomal cargo uptake is correlated with the exosome dosage. (B) Podocytes were treated with Cy3-miRCtrlExos for 4, 12, 24 and 48 hours followed by staining for CD9. The exosomal cargo uptake by podocytes can already be seen at 4 h and increases over time until 48 hours. F-Actin is stained with phalloidin. Nuclei are stained with DAPI. Scale bars = 20 µM.

Supplementary Figure 5: Exosomes loaded with Pxn-siRNA downregulate Pxn expression in podocytes. Podocytes were treated with siRNACtrlExos or Pxn-siRNAExos for 48 hours. The Pxn fluorescence decreased in the Pxn-siRNA Exo-treated samples in almost every cell. F-Actin was stained with phalloidin. Scale bars = 20 µm.
